# Analysis of Donor Motivations in Living Donor Liver Transplantation

**DOI:** 10.3389/fsurg.2014.00025

**Published:** 2014-07-08

**Authors:** Hesham Abdeldayem, Samy Kashkoush, Bassem Soliman Hegab, Amr Aziz, Hany Shoreem, Shereef Saleh

**Affiliations:** ^1^National Liver Institute, Menofeyia University, Cairo, Egypt

**Keywords:** living donor, LDLT, motivations, altruism, coercion, ethics

## Abstract

**Objectives:** The introduction of the living donor liver transplantation (LDLT) in Egypt as in elsewhere, has raised important psychological conflicts and ethical questions. The objective of this study was to get better understanding of the potential donors’ motives toward LDLT.

**Methods:** This study was conducted on consecutive 193 living-liver donors who underwent partial hepatectomy as donors for LDLT during the period between April 2003 and January 2013, at the National Liver Institute Menoufeyia University, Egypt. Potential donors were thoroughly evaluated preoperatively through a screening questionnaire and interviews as regard their demographic data, relationship to the potential recipient, and motives toward proceeding to surgery. They were assured that the information shared between them and the transplant center is confidential.

**Results:** The donors’ mean age was 25.53 ± 6.39 years with a range of 18–45 years. Males represented 64.7% and females were 35.3%. The most common donors (32.1%, *n* = 62) were sons and daughters to their parents (sons: *n* = 43, daughters: *n* = 19) while parents to their offsprings represent 15% (mothers: *n* = 21, fathers: *n* = 8). Brothers and sisters represent 16.5% (brothers: *n* = 22, sisters: *n* = 10). Nephews and nieces giving their uncles or aunts were 14%. The number of wives donating to their husbands was 11 (5.7%). Interestingly, there was no single husband who donated his wife. Among the remaining donors, there were 11 cousins and 1 uncle. Unrelated donors were 20 (10.4%). Several factors seemed to contribute to motivation for donation: the seriousness of the potential recipient condition, the relationship and personal history of the donor to the potential recipient, the religious beliefs, the trust in the health care system, and family dynamics and obligations.

**Conclusion:** Absolute absence of coercion on the living-liver donor’s motives may not be realistic because of the serious condition of the potential recipient. It is mandatory that the donor is truly willing to donate.

## Introduction

The concept of living donor liver transplantation (LDLT) has allowed the introduction of liver transplantation in Egypt, as well as, in other countries where deceased donor liver transplantation is not possible (1, 2). Habib et al. performed the first LDLT in Egypt, in 1991, at the National Liver Institute (3). The number of LDLT operations has increased in the past few years and in January, 2014, the number of cases in Egypt exceeded 2000.

Risks to the donor are the most important concerns in LDLT. Autonomy indicates that individuals should decide what sort and amount of risk they are willing to face (4). For potential donors, LDLT represents the only means to save the lives of their loved ones. However, the very short time and the lack of alternative treatment, the impact of moral guides, the social circumstances, and the possibility of complications constitute high pressure and considerable amount of coercion on the donor’s will. This assumption would add to the ethical considerations regarding the motives of the donor (5, 6).

## Materials and Methods

This study was conducted on consecutive 193 living-liver donors who underwent partial hepatectomy for LDLT, during the period between April 2003 and January 2013 at the National Liver Institute Menoufeyia University, Egypt. All donors were interviewed. During the interview, it was made clear that participation would be confidential and would therefore not affect any treatment they or their loved ones were currently receiving. Each interview lasted for approximately 30 min. Two reviewers assessed each case independently and resolved any disagreement by discussion. The interviews were structured, using mainly open questions, encouraging the donors to express themselves freely and reflect on their intentions to donate. The questionnaire were reproduced from a study made by Papachristou et al. (5). Open questions were used. The potential donors were encouraged to express their intentions for donation (5).

### Questionnaire

The questions given to the potential donors are designed to assess their attitudes, views, and motives to LDLT. Potential donors were asked about their willingness to become living related donors, their trust in hospitals, and their feelings about religion. They were asked about their age, gender, education, marital status, dependents, and comorbid conditions. The aims of the questionnaires were to verify their emotional relations to the recipient, to revise their consent, to exclude coercion, to assess their social and family backgrounds, and to exclude any form of trade particularly for unrelated donors.

We classified factors associated with willingness to donate into: demographic factors (age, gender and occupation), relationship to the recipient, socioeconomic factors (marital status presence and number of dependents, level of education, employment status, income, health insurance status), and religious attitude. Concerns regarding the surgical procedure were recorded and included: the risk of complications from the procedure, the hospital length of stay, the amount of compensated and uncompensated time from work, the need for narcotic pain medications, and the risk of recipient liver failure and death.

### Data analysis

The interviews were initially evaluated case by case and then analyzed comparatively. Because of the lack of standardized means to examine living donors’ motivation, qualitative methodology was considered the suitable means for describing and analyzing such complex issues of psychosocial or psychological natures. Thus, the analysis of the interview data aimed at verifying, not quantifying, motivational attitudes of the potential donors. Findings are not expected to be statistically projectable.

Reproduced from the previously mentioned study made by Papachristou et al. (5) we classified our potential donors into:

1. The altruistic donor: the well-being and the life of the recipient are of utmost priority.
2. The relationship-oriented donor: the emotional donor–recipient relationship and its maintenance are the main motives for donation.
3. The moral donor: ethical principles are of high priority to the donor. Religious or spiritual background is of great importance.
4. The self-interested donor: the donor’s feelings, expectations, and personal profit are of priority. The donation is an attempt to take control over a stressful situation, to reduce anxiety and fear of loss. The maintenance of the self-image is a strong motivation. The donation may be seen as a personal challenge.
5. The ambivalent donor: the motivation for donation is not clear. The relationship to the recipient is controversial, and the advantages and disadvantages of the surgery cannot be estimated.

## Results

### Demographic data

- Age: the donors mean age was 25.53 ± 6.39 years with a range of 18–45 years.
- Gender: males represented 64.7% and females were 35.3%.
- Occupation: college students (*n* = 25), farmers (*n* = 22), teachers (*n* = 19) office assistants (*n* = 18), medical personnel (*n* = 17), laborers (*n* = 17), engineers (*n* = 10), lawyers (*n* = 8) others (*n* = 23), unemployed (*n* = 25).

Those more likely to proceed for donation were men rather than women (64.7 vs. 25.3%), young age (76% aged 18–30 years vs. 24% aged 30–45 years), more educated (62% are high college students or have university degree vs. 38% have no formal qualifications), and single rather than married (73 vs. 27% respectively). Unrelated donors donating to a close friend (*n* = 20) tend to be of younger age (20–29 years) and married (15 out of 20 donors).

### Donors’ relation to recipient

- Child generation (*n* = 62) 32.1% (sons were 43 and daughters were 19).
- Parents generation (*n* = 29) 15% (mothers were 21, father were 8).
- Nephews and nieces giving their uncles or aunts were 14% (*n* = 27).
- Sibling: brothers and sisters represent (*n* = 32) 16.5% (sisters were 10 and brothers were 22).
- Cousins (*n* = 11).
- Spouses: wives donating to their husbands were 11 (5.7%). Interestingly, there were no single husbands who donated his wife.
- One uncle.
- Unrelated donors were 20 (10.4%).

### Recipient diseases

While the number of donors for adult recipients was 164, the number for pediatric recipients was 29. For all donors, LDLT represented the only available life-saving maneuver for their recipients. In 15 cases, the decision had to be made urgently because of the urgency of the recipient’s status.

### Donor–recipient relationship

Interestingly, all donors stated that their relationship with their recipients is very stable, very close, and very good.

### Importance of religion in making up the donor decisions

Interestingly, all donors expressed the importance of their religious beliefs in their decision. Of them, 172 were Moslems and 21 were Christians (Coptic Church).

### Fear of the operation

About half of the potential donors (*n* = 97) stated that they do not have any fears of the donation part of their liver to their loved ones. On the other hand, about 45.5% (*n* = 88) claimed mild amount of fears, and only 4.5% (*n* = 8) donors have admitted that they have great fears over the donation process.

### Motives for donation

One hundred and seventy donors (about 88%) indicated their firm determination to donate “*openly motivated donors*.” They showed absolute willing to donation, and wanted to go for surgery as quickly as possible to save the lives of their recipients. They stated reasons in favor of, and none against donation. They considered the risk faced by the surgery as very low and no or mild fears. The interview tends to be short. About 72% (*n* = 151) answered in short sentences, just expressing their altruistic willing, without mentioning any details about their personal motivations or concerns.

Only 23 donors (about 12%) admitted their concerns about the procedure. They initially stated that they had not decided yet “*openly ambivalent donors*.” They mentioned their reasons against the operation as: the major risks to the recipient (*n* = 10), the possibility of serious complications to the donor (*n* = 8), and the lack of trust in health system (*n* = 5).

Almost all donors were aware of family expectations, even if they were not mentioned clearly by the members of the family. Four sibling donors admitted that they had tight familial obligation and were willing to donate to avoid stresses within the family. The concept of accepting the recipient’s ill-heath was unbearable for considerable number of donors (*n* = 62). In 19% of cases (*n* = 37), donors have in the past, stressful feelings of loss of one or more of their beloveds, e.g., a parent. In cases where the parents were donating to their sons or daughters, all donor parents (*n* = 29) argued that their children are too young to die and that they have not yet enjoyed their lives. Interestingly, in 10 donors (5.2%), the motive for donation was not purely a method to save the lives of their recipients. For them, the main motive was the eagerness to undertake an outstanding, risky experience. Nine donors (4.7%) expressed that their religious and moral principles and beliefs were the main motive to donate. For them, donation held meaningful ethical aspects. Seven donors (3.7%) admitted that, if they refuse proceeding to donation that may affect their self-image and self-esteem and will result in feelings of guilt. For six donors (3.1%) the motive for donation was to protect younger members in the family from pains that they would suffer if the recipient dies, for whom his or her existence is invaluable.

## Discussion

Current guidelines recommend that living-liver donors should be free from “coercion.” However, it is argued that the complete absence of coercion or obligation is unrealistic because the act of donation is “life saving” or perceived to be the only available treatment option (2, 7). Every effort should be paid to verify that the donation offered by the potential living-liver donor is genuine, voluntary, and free from coercion. He or she should be of suitable age, competent, and medically free, fully informed about the potential risks, benefits, and availability of any alternative therapy to the potential recipient and clearly willing to donate (7, 8). Every effort should be paid to ensure that the potential donor understands clearly the risks of complications and death due to surgery for both the donor and the recipient. Ensuring that the donor decision to go on for donation is free and not as a result of any sort of coercion, which is a very difficult task (7, 9).

Someone may argue that some potential living-liver donors may feel that they are under pressure to donate to their beloved recipients and they are incapable of having freedom of choice. On the other hand, some others would argue that moral values and emotional feelings do not interfere with freedom to decide but are rather a part of the human life. In all circumstances, the donor should be given an enough time to review his or her decision and should be offered the chance to withdraw at any point before the procedure (4, 10).

Three factors determine a donor’s motivation concerning living-donor liver transplant: the relational and the emotional bonds between the donor and the recipient couple, the socioeconomic background of the donor, the likely benefits gained by the donor from donation, and the personal attitude toward donation (11). These factors are dynamically interrelated and influence the donor’s motivation.

The ethical prerequisite for accepting persons as living-liver donors is the “autonomy” (11, 12). Autonomy represents the basis of the donors’ free informed consent. The transplant team should provide the potential donor with clear, detailed, and unbiased information regarding the procedure. On basis of this information, the potential donor will make up his or her mind (1, 11). The donor should be allowed to express openly all of his or her concerns, anxieties, and fears (11–13).

This study was designed to report the own words and expressions of the potential living-liver donors thus allowing better understanding of how do they make up their minds and their willingness and motivations toward donation (13, 14). The aim of our study was to highlight the interactions and the interrelations of the autonomy and motives besides all information as regards to risks and benefits of LDLT in the potential donor’s final decision to donate. Assessing concepts like: decision making, coercion, emotions, relative benefits, and fears is so difficult (1, 11). We classified living-liver donors into two extreme types: “the openly ambivalent donors” and “the openly motivated donors.”

Willingness for living-liver donation is affected mainly by the potential donor emotions and relationship toward the potential recipient. Openly motivated donors as a rule express tendency to idealize their emotional relationship. Openly ambivalent donors mainly express concerns related to their fears about the procedure, particularly the risks of complications and death (13–15). LDLT represents to many donors the only chance to save the lives of their beloved ones. They consider donation as an automatic response and obligation to do whatever is possible to actively help them (13). Some living-liver donors state that they are scared of how the LDLT surgery will affect their own lives, but helping their loved ones is a more important significant issue. Saving the patient life will help the donor too, as they will no longer have the tension of seeing them suffering and dying. The donor knows that donation will be of benefit to both the patient and donor him- or herself (1, 14). A clear example is the self-benefit from improvement of a couple’s quality of life when a spouse considers donation to his or her companion (1, 15).

The donor’s feeling that, the transplant team will not perform the LDLT procedure unless they are sure of the good outcomes, reassures the donor. This belief may be translated as redirecting much of the responsibility for the donation decision onto the transplant surgical team (4, 14).

Several factors support the motives of the potential living-liver donor to donate. They include: previous awareness of donation and the transplant procedure. Knowledge of the patient’s health status and future need of a LDLT, trust in the transplant team and family support (4, 10, 16). On the other hand, there are factors that may raise donors’ concerns. These factors include: fear of being medically unfit for donation, fear of poor outcome or death of the recipient, fear of long-term complications of the procedure, unstable emotional relationship with the recipient, and objections within the family (1, 16).

In the Egypt, the law considers the sale of organs as an illegal act. A proof of a relationship between potential donor and patient must be provided before the procedure (1, 2). However, regardless of the strict rules, it is sometimes impossible to assure that a potential living-liver donor meets the prerequisites (17). Friends as living-liver donors may feel less obligated to donate if compared to family members. They may experience greater satisfaction and increased self-esteem without being coerced by any sense of obligation (4).

As for living unrelated donation by strangers, the authors express doubts about their real motives to donation, their realization of the risks of complications and death to the donor, their psychological and emotional stability, and the possibility of future financial requests from the recipient’s family.

## Conclusion

In LDLT, a purely autonomous motivation of the potential living-liver donor, a clearly altruistic attitude, or an absolute absence of coercion, may be impossible to prove. Our study describes the factors involved in the motivations for the decision-making in LDLT. Several factors appear to contribute: the seriousness of the potential recipient health condition, the strength of the relationship and personal history of the donor toward the potential recipient, the religious beliefs, the trust in the health care system, and the family obligations. Strategies that aim to safeguard against unwarranted coercion are needed to ultimately protect the safety and well-being of living liver donors.

## Conflict of Interest Statement

The authors declare that the research was conducted in the absence of any commercial or financial relationships that could be construed as a potential conflict of interest.
