# Corrigendum: Analysis of donor motivations in living donor liver transplantation

**DOI:** 10.3389/fsurg.2020.00034

**Published:** 2020-06-30

**Authors:** Hesham Abdeldayem, Samy Kashkoush, Bassem Soliman Hegab, Amr Aziz, Hany Shoreem, Shereef Saleh

**Affiliations:** National Liver Institute, Menofeyia University, Cairo, Egypt

**Keywords:** living donor, LDLT, motivations, altruism, coercion, ethics

In the original article, there was an error. Although the publication by Christina Papachristou et al. 2004 was referred to within the text and in the Reference section, the authors would like to rephrase some sentences in the section of The Materials and Methods and The Data Analysis subsection to give proper credit to the above mentioned work and to make it clear that some of the information was reproduced from this work and to insert a reference in the proper place.

A correction has been made to the **Materials and Methods** section, paragraph 1:

“This study was conducted on consecutive 193 living-liver donors who underwent partial hepatectomy for LDLT, during the period between April 2003 and January 2013 at the National Liver Institute Menoufeyia University, Egypt. All donors were interviewed. During the interview, it was made clear that participation would be confidential and would therefore not affect any treatment they or their loved ones were currently receiving. Each interview lasted for approximately 30 min. Two reviewers assessed each case independently and resolved any disagreement by discussion. The interviews were structured, using mainly open questions, encouraging the donors to express themselves freely and reflect on their intentions to donate. The questionnaire were reproduced from a study made by Papachristou et al. (5). Open questions were used. The potential donors were encouraged to express their intentions for donation (5).”

A correction has been made to the **Materials and Methods** section, subsection **Data Analysis**, paragraph 2:

“Reproduced from the previously mentioned study made by Papachristou et al. (5) we classified our potential donors into:

1. The altruistic donor: the well-being and the life of the recipient are of utmost priority.
2. The relationship-oriented donor: the emotional donor–recipient relationship and its maintenance are the main motives for donation.
3. The moral donor: ethical principles are of high priority to the donor. Religious or spiritual background is of great importance.
4. The self-interested donor: the donor's feelings, expectations, and personal profit are of priority. The donation is an attempt to take control over a stressful situation, to reduce anxiety and fear of loss. The maintenance of the self-image is a strong motivation. The donation may be seen as a personal challenge.
5. The ambivalent donor: the motivation for donation is not clear. The relationship to the recipient is controversial, and the advantages and disadvantages of the surgery cannot be estimated.”

The authors apologize for this error and state that this does not change the scientific conclusions of the article in any way. The original article has been updated.

